# Development of one-step multiplex real-time PCR for the detection of CHV-1, CAdV-2, and CDV

**DOI:** 10.3389/fvets.2025.1583769

**Published:** 2025-05-09

**Authors:** Yifan Li, Jingqi Wu, Chaonan Wang, Zheng Jia, Zaixing Yang, Wei Lin, Junwei Ge, Lili Zhao

**Affiliations:** ^1^State Key Laboratory for Diagnosis and Treatment of Severe Zoonotic Infectious Diseases, Key Laboratory for Zoonosis Research of the Ministry of Education, Institute of Zoonosis, and College of Veterinary Medicine, Jilin University, Changchun, China; ^2^College of Veterinary Medicine, Northeast Agricultural University, Harbin, China

**Keywords:** multiplex real-time PCR, CIRDC, canine herpesvirus-1, canine adenovirus-2, canine distemper virus

## Abstract

Canine Infectious Respiratory Disease Complex (CIRDC) is a highly contagious disease that frequently affects canine populations and has emerged as a global epidemic. It has been reported that CIRDC can have a serious impact on related life. Therefore, the rapid detection and differentiation of common viruses that cause CIRDC are essential. It is generally believed that CIRDC is mainly caused by infection of three pathogens: canine herpesvirus-1 (CHV-1), canine adenovirus-2 (CAdV-2), and canine distemper virus (CDV). In this study, we developed and validated a TaqMan probe-based multiplex real-time PCR method to detect and identify these three viruses simultaneously. We designed specific primers and probes, and optimized the concentrations of each reactant in the system. The method was found to have good sensitivity, specificity and stability, and had a limit of detection of 10^2^ copies/μL, 10^1^ copies/μL and 10^1^ copies/μL for CHV-1, CAdV-2, and CDV, respectively. In addition, co-infection simulation experiments confirmed that the method worked effectively, even if the concentrations of multiple viruses in the sample were close to the limit of detection or the concentrations of different viruses were different. The method was used to detect 122 clinical samples, and the results showed that it was more sensitive and reliable than conventional singleplex PCR. Thus, the method developed in this study is suitable for the clinical monitoring of CIRDC and is of great significance for the prevention and management of respiratory diseases in canine populations.

## 1 Introduction

Canine Infectious Respiratory Disease Complex (CIRDC) is a contagious disease involving multiple pathogens, and outbreaks have been reported all around the world (1–4). CIRDC has been referred to historically as “canine infectious tracheobronchitis” and is described as an acute, highly contagious respiratory infection in dogs (5). Diseased dogs commonly exhibit a range of respiratory symptoms, including coughing, nasal discharge, and difficulty breathing. In most cases, these symptoms resolve spontaneously within a short period of time; however, in cases of co-infection or immunosuppression, some dogs develop severe bronchopneumonia, which can be fatal (6, 7). Due to the high contagiousness and risk of death associated with CRIDC, the occurrence of disease is extremely detrimental to boarding kennels, animal shelters, and training classes for working dogs as it leads to significant economic losses and management challenges. Thus, early diagnosis is essential for the timely isolation, treatment, and control of disease. CIRDC is caused by several pathogens, including canine adenovirus-2 (CAdV-2), canine distemper virus (CDV), canine herpesvirus-1 (CHV-1), among others (7, 8). Some studies have shown that co-infections with the three pathogens often occurs in CIRDC (7, 9–11), with a co-infection rate of 9.8% in one study of clinically symptomatic dogs (10). Co-infection can lead to more severe clinical symptoms compared to single infections (12). Therefore, the establishment of multiplex detection methods for the three CIRDC-related viruses has attracted increasing attention.

CAdV is non-enveloped double-stranded, linear DNA virus belonging to the *Adenoviridae* family and the genus *Mastadenovirus*, and is divided into canine adenovirus type 1 (CAdV-1) and 2 (CAdV-2) based on the serotypes (13, 14). The two known serotypes display 75% identity at the nucleotide level (15), but differ in their pathogenicity and cell tropism, which may be due to differences in the gene coding the fiber protein in the two types of CAdV (16). CAdV-2 is typically restricted to infections of the upper respiratory tract (17). However, there are also reports that CAdV-2 can cause severe necrotizing bronchitis and interstitial pneumonia (18). It is also associated with fatal cases of diarrhea (19) and neurological diseases in dogs (20). In addition, outbreaks of CAdV-2 have been reported across the globe, including in recent studies (21, 22).

CDV is enveloped single-stranded RNA viruses, belonging to the *Paramyxoviridae* family, genus *Morbillivirus* (23). The RNA encodes six structural proteins, including two membrane glycoproteins, the fusion (F) and hemagglutinin (H) proteins, the envelope-associated matrix (M) protein, the phosphoprotein (P), the large polymerase (L), and the nucleocapsid (N) protein. Among those proteins, the H and F genes display a high degree of nucleotide variation that allows for the distinction of several genotypes or genetic clusters (24), whereas the N protein-encoding gene is subjected to a lower degree of variation (25). CDV infects dogs and many other domestic and wild carnivores and initially replicates in lymphoid cells of the respiratory tract leading to immunosuppression (26). The virus then disseminates throughout the body 3 ∼ 4 days after infection, multiplying in epithelial cells of different organs (respiratory, gastrointestinal, skin) (27). Occasionally, the virus invades the central nervous system (CNS), inducing severe neurological disease or even death (28). In addition, infection of multiple carnivores has occurred throughout the world (29).

CHV-1 is enveloped DNA viruses belonging to the family *Herpesviridae* and was first identified as a canine pathogen in 1965 (30). CHV-1 has multiple coding genes, such as glycoprotein (g) genes gB, gC, gD, gH, and the thymidine kinase gene, among others. Among the proteins, gB is often used as a PCR target for CHV-1 detection due to its high level of conservation and specificity (31–33). The host range of CHV-1 is restricted to canids. Because infection causes respiratory symptoms, CHV-1, along with CDV and CAdV-2, is considered the main pathogen associated with CIRDC (21, 34). In neonatal puppies (1–2 weeks of age), systemic infection with CHV-1 is fatal and may affect the entire litter (35). In puppies over 2 weeks of age and in adult dogs, CHV-1 can establish latent infections that can be readily reactivated by immunosuppression (36). In some individuals, death from a single infection has occurred (37). In addition, the virus has a worldwide distribution, with more than 80% seroprevalence in some dog populations (38).

CIRDC is typically caused by co-infection with multiple pathogens, and the clinical symptoms caused by each virus are similar and not easy to distinguish. Thus, to prevent outbreaks, there is a need for rapid and sensitive diagnostic tools for early differential diagnosis of co-infections. Virus isolation is regarded as the gold standard for CIRDC virus detection (17, 39, 40), however, that method is characterized by prolonged detection times and low success rates. Ongoing research in the field of immunology continues to explore related areas. Enzyme-linked immunosorbent assay (ELISA) for antibodies against these three viruses is also considered an effective detection method (41–44). However, ELISA is complex and is not suitable for large-scale monitoring. In addition, a variety of molecular biology detection methods for CIRDC viruses have been established, such as conventional PCR (31, 45, 46), nested PCR (47), and SYBR Green qPCR (48). However, these single detection methods require separate amplifications of each pathogen, which is cumbersome and time-consuming, and is not suitable for CIRDC which is prone to co-infection. Multiplex PCR detection methods developed in some studies also suffer from low sensitivities (10). There is currently no effective multiplex method for detecting the three pathogens in the same reaction.

In this study, we developed and evaluated a multiplex real-time PCR method based on TaqMan probes for detecting the three CIRDC-related viruses. The method enabled the simultaneous detection of each virus by using three sets of specific primers and probes in a single reaction mixture, exhibiting revolutionary potential for its application in clinical diagnosis. The assay performance was verified evaluations of its specificity, sensitivity, repeatability, and detectability in clinical samples. Consequently, this method enabled rapid and accurate identification and detection of CAdV-2, CDV, and CHV-1, which is significant for the early prevention and control of CIRDC.

## 2 Materials and methods

### 2.1 Primer and probe design

To ensure the optimal performance of the primers and probes used in the multiplex real-time PCR, those for CHV-1 were referenced from prior reports using the gB-encoding gene as the target (49). The primers and probes for the two viruses were designed using conserved sequences. The available sequences for the fiber gene of CAdV-2 and the N gene of CDV from GenBank were obtained and aligned using DNAMAN (LynnonBiosoft, USA) software to identify the highly conserved regions of each gene. Then, primers and probes were designed to target the conserved regions using the Primer Premier 5 program, and filtered based on the design criteria such as hairpin configurations and primer dimer formations. The specificity of the filtered primers and probe sequences were validated in silico using NCBI Basic Local Alignment Search Tool (BLAST)^1^. In addition, the primers of the three viruses for conventional PCR in this study were, respectively, referred to the following sources: The CHV primers were obtained from prior report (47) and the CAdV-2 primers were obtained from the Chinese Experimental Journal Group Standard of the Physical Society (T/CALAS 68–2019), CDV primers were obtained from the National Standard of the People’s Republic of China (GB/T 27532–2011). All primers and probes were synthesized by Comate Bioscience (Changchun) Co., Ltd., and the details are shown in Table 1.

**Table 1:** Primer and probe sequences.

Types of PCR	Primer or Probe name	Sequence (5′-3′)	Length (bp)	Position	GenBank accession
Real-time PCR primers and probes	CHV-1-DF	ACAGAGTTGATTGATAGAAGAGGTATG	136	439–574	No. AF361073
	CHV-1-DR	CTGGTGTATTAAACTTTGAAGGCTTTA			
	CHV-1-Probe	Cy5-TCTCTGGGGTCTTCATCCTTATCAAATGCG-BHQ1			
	CAdV-2-DF	GTTTTGTCTTTTACCTCCCCATT	83	26,886–26,968	No. U77082.1
	CAdV-2-DR	CCATTTTCATCTTCTAACCCATC			
	CAdV-2-Probe	ROX-TAGCGCTAGGGATACAGTGTTTTCATT-BHQ2			
	CDV-DF	GGCACTCATCTTGGACATCA	104	809–912	No. MF926604.1
	CDV-DR	AACTAGCTAACCCAGCTTCC			
	CDV-Probe	FAM-TTCAGCAATACTAGGCTTGTTCCCTGG-BHQ1			
Gel-based PCR primers	CHV-1-GF	TGCCGCTTTTATATAGATG	493	283–776	No. X75765.1
	CHV-1-GR	AAGCGTTGTAAAAGTTCGT			
	CAdV-2-GF	CGCGCTGAACATTACTACCTTGTC	505/1030	25,406–25,910	No. NC_001734.1
	CAdV-2-GR	CCTAGAGCACTTCGTGTCCGCTT			
	CDV-GF	CGAGTCTTTGAGATAGGGTT	455	7,813–8,267	No. MK037467.1
	CDV-GR	CCTCCAAAGGGTTCCCATGA			

### 2.2 Virus strains and field samples

Clinical samples collected during 2019–2022 were preserved at −80°C in our laboratory. Those samples, including nasopharyngeal swabs, feces, blood, and tissue samples (lymph nodes, spleen, liver) were primarily collected from pet hospitals, pet markets, and breeding bases in the three northeastern provinces of China. Samples were then identified by conventional singleplex PCR and confirmed by DNA sequencing at Comate Bioscience (Changchun) Co., Ltd. Among the samples, there were samples positive for five strains, including CDV, Canine parainfluenza virus (CPV), Canine Coronavirus (CCoV), Canine Astrovirus (CAstV), and Canine Chaphamaparvovirus (ChPV). For specific information, please refer to previous studies (50). CAdV-2 and Canine parainfluenza virus (CPIV) were derived from the purchased canine Vanguard® Plus 5 vaccine (CPIV is NL-CPI-5 strain; CAdV-2 is Manhattan strain). All positive samples were properly labeled and stored at −20°C.

### 2.3 Nucleic acid extraction and reverse transcription

Viral DNA or RNA was extracted using the EasyPure Viral DNA/RNA Kit (Beijing TransGen Biotech Co., Ltd.) according to manufacturer’s instruction. Viral nucleic acids were eluted in nuclease-free water. Reverse transcription was performed using the ReverTra Ace® qPCR RT Master Mix (Beijing Huaruikang Technology Co., Ltd.).

### 2.4 Construction of plasmid standards

The target fragments of CAdV-2, and CDV were amplified separately via with the Ex Taq® PCR Enzyme (Beijing Takara Biomedical Technology Co., Ltd.) using the DNA/cDNA obtained in the previous step. The primers for CAdV-2 and CDV were the same as those in prior reports (7, 51). For the CHV-1 plasmid standard, the genes were synthesized by Comate Bioscience (Changchun) Co., Ltd. based on previous studies (49). PCR products were cloned into the pMD-19 T vector (Beijing Takara Biomedical Technology Co., Ltd.) and confirmed by DNA sequencing. The plasmid copy number was calculated and the plasmids were diluted from 1 × 10^7^ copies/μL to 1 × 10^2^ copies/μL. Singleplex real-time PCR was performed for each virus using the 10-fold serial dilutions of the plasmid standards to generate standard curves, based on which the *R*^2^ (correlation coefficient), *E* value (amplification efficiency), and standard equations were calculated.

### 2.5 Reaction conditions of the singleplex real-time PCR assay

The singleplex real-time PCR assay was 20 μL, consisting of 10 μL of Premix Ex Taq from qPCR Probe Master Mix kit (Beijing Takara Biomedical Technology Co., Ltd.), 0.5 μL of each primer (10 μM), 0.5 μL of TaqMan probe (10 μM), 2 μL of template, and the remaining volume of nuclease-free water. Amplification was carried out on a CFX96 fluorescence quantitative PCR instrument (Bio-Rad Laboratories) using the following program: 95°C for 5 min; 40 cycles of 94°C for 35 s, and 60°C for 35 s. The fluorescence signal was automatically collected at the end of each cycle.

### 2.6 Reaction condition optimization for multiplex real-time PCR

The singleplex real-time PCR assays for CHV-1, CAdV-2, and CDV described above were multiplexed into one reaction consisting of Premix Ex Taq from the qPCR Probe Master Mix kit (Beijing Takara Biomedical Technology Co., Ltd.), primers and probes for all three viruses, and templates. The multiplex reaction was then optimized using different annealing temperatures, different volumes of primers (10 μM) and probes (10 μM), and different volumes of templates. During the optimization stage, the final concentration of primers and probes ranged from 50 nM to 500 nM. The plasmid standards containing 1 × 10^5^ copies/μL were chosen as the templates. The same instrument and real-time PCR program were used as described above.

### 2.7 Sensitivity of the multiplex real-time PCR assay

To determine the limit of detection of the multiplex assay, we performed real-time PCR for each virus separately, using 10-fold serial dilutions of the standard plasmid templates, ranging from 1 × 10^7^ copies/μL to 1 × 10^0^ copies/μL. To confirm the limit of detection, multiplex real-time PCR was performed using plasmid templates for all three viruses at the estimated limit of detection, with 25 replicates for each concentration. The lowest concentration that met the positive detection rate of 95% was considered as the reliable limit of detection.

### 2.8 Specificity of the multiplex real-time PCR assay

To rule out potential false positives caused by other viruses that may be present in the samples, samples positive for CHV-1, CAdV-2, CDV, CPV, CCoV, CPIV, CAstV, and ChPV were tested using the multiplex real-time PCR assay. All DNA/cDNA samples were previously synthesized and stored in our laboratory.

### 2.9 Repeatability of the multiplex real-time PCR assay

To test the stability of the multiplex real-time PCR method, separate assays were performed to detect the standard plasmids at different concentrations and compare the Cq values. Three different concentrations of positive samples were used as templates. Three batches of tests were performed, with each sample run in triplicate. Intra-group and inter-group repeatability results were used to calculate the coefficient of variation (C.V.).

### 2.10 Simulation of co-infection by combining different plasmid standards

Plasmid standards of two target pathogens at the same concentration were randomly chosen and mixed as templates and detected using the new method. Two concentrations (limit of detection and 10 times the limit of detection) of the plasmid standards were tested. To simulate triple co-infection events, we mixed the plasmid standards of the three target pathogens with one at 1 × 10^7^ copies/μL and the other two at 10 times the limit of detection and detected the template mixture using the multiplex detection method.

### 2.11 Clinical sample detection

We tested 122 samples collected from canines, including those with respiratory symptoms and those without any clinical symptoms. The clinical performance of the method was evaluated by comparing the detection rates to those of conventional singleplex PCR. Regarding the specific methods of conventional PCR for the three viruses in this study, refer to the sources mentioned in 2.1 above, respectively, and make slight modifications. These PCR were conducted in a 20 μL reaction containing 1 μL of primers (10 μM) (Table 1), 2 μL of PCR Buffer, 2 μL of dNTP mixture, 0.2 μL of the Ex Taq® PCR Enzyme (Beijing Takara Biomedical Technology Co., Ltd.), and 1 μL of template DNA. The cycling protocol for CHV-1 consisted of preheating at 94°C for 3 min following by 40 cycles of 94°C for 30 s, 53°C for 30 s and 72°C for 1 min, with a final extension at 72°C for 10 min. The cycling protocol for CAdV-2 consisted of preheating at 95°C for 5 min following by 35 cycles of 94°C for 30 s, 62°C for 30 s and 72°C for 70s, with a final extension at 72°C for 10 min. The cycling protocol for CDV consisted of preheating at 94°C for 2 min following by 35 cycles of 94°C for 30 s, 55°C for 30 s and 72°C for 40s, with a final extension at 72°C for 3 min. The PCR products were detected by electrophoresis in 1.5% agarose gel and visualized under UV light after ethidium bromide staining.

### 2.12 Statistical analysis

The plasmid standards data are expressed in copies/μL (template concentration). Means and standard deviations were calculated using GraphPad Prism software version 8.0 (Graph-Pad, Inc., La Jolla, CA, USA). The coefficient of variation (%CV) was calculated using the following formula: %CV = 100 × (standard deviation of replicates [log10 copies/μL] ÷ average of replicates [log10 copies/μL]).

## 3 Results

### 3.1 Preparation of primers, probes and plasmid standards

**Figure 1:**
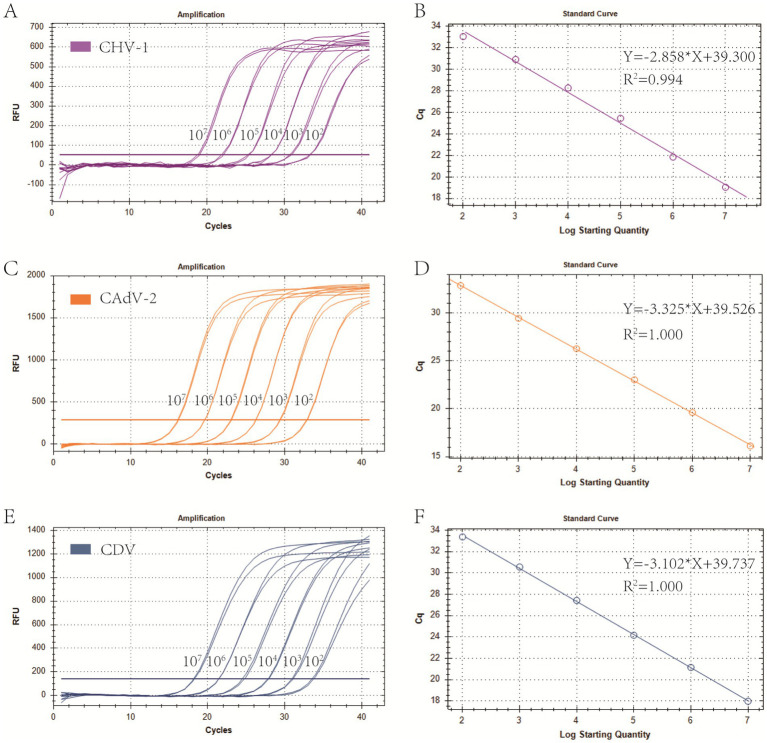
Preparation of plasmid standards. **(A,C,E)** Amplification curves (X-axis: Cycle, Y-axis: Fluorescence) of CHV-1, CAdV-2, and CDV for each plasmid with concentrations of 1 × 10^7^ copies/μL to 1 × 10^2^ copies/μL. **(B,D,F)** Standard curves of CHV-1, CAdV-2, and CDV plasmids.

Primers and probes designed in this study were provided in Table 1. Plasmid standards with concentrations ranging from 1 × 10^7^ copies/μL to 1 × 10^2^ copies/μL of each pathogen were selected to perform singleplex real-time PCR (Figure 1). The standard curves showed an acceptable *R*^2^ and *E* values: CHV-1 *R*^2^ = 0.994, *E* value = 109.8%; CAdV-2 *R*^2^ = 1.000, *E* value = 99.9%; and CDV *R*^2^ = 1.000, *E* value = 109.2%, indicating that the plasmid standards were qualified, and the primers and probes designed were efficient.

### 3.2 Optimization of the multiplex reaction system

**Figure 2:**
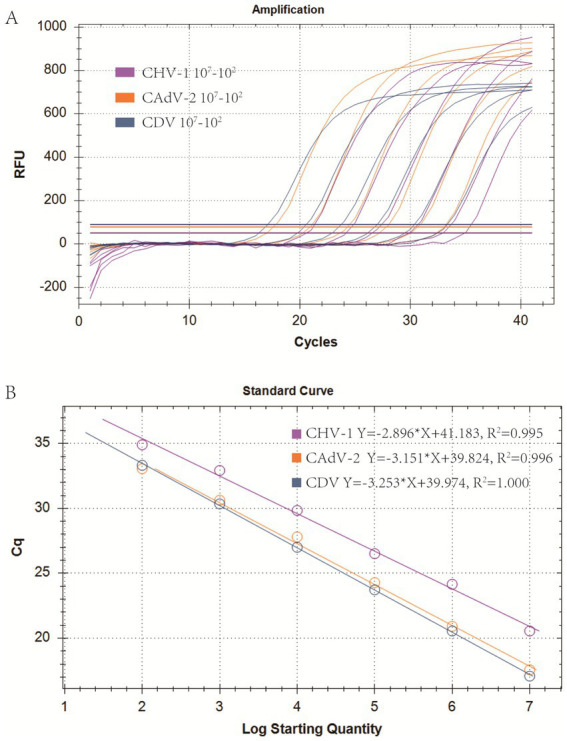
Optimization of the multiplex real-time PCR system and establishment of standard curves. **(A)** Amplification curves (X-axis: Cycle, Y-axis: Fluorescence) of CHV-1, CAdV-2, and CDV for each plasmid with concentrations of 1 × 10^7^ copies/μL to 1 × 10^2^ copies/μL in multiplex real-time PCR. **(B)** Standard curves of CHV-1, CAdV-2, and CDV plasmids in the multiplex real-time PCR assay.

Real-time PCR of four viruses was optimized by determining the concentrations of probes and primers. The singleplex real-time PCR for CHV-1, CAdV-2, and CDV was combined into one reaction system. The optimized primer and probe additions were: 0.8 μL of the CHV-1-DF/CHV-1-DR primer set (10 μM), 0.8 μL of the CHV-1-Probe (10 μM), 0.3 μL of the CAdV-2-DF/CAdV-2-DR primer set (10 μM), 0.3 μL of the CAdV-2-Probe (10 μM), 0.3 μL of the CDV-DF/CDV-DR primer set (10 μM), and 0.3 μL of the CDV-Probe (10 μM). The annealing temperature was optimal at 60°C. Using the optimized reaction system and plasmid standards with six concentration gradients from 1 × 10^7^ copies/μL to 1 × 10^2^ copies/μL, the standard curves for multiplex real-time PCR were established. The standard curve for each virus is shown in Figure 2 and the parameters of each standard curve were as follows: CHV-1 (*Y* = −2.896*X + 41.183, *R*^2^ = 0.995, *E* = 108.5%), CAdV-2 (*Y* = −3.151*X + 39.824, *R*^2^ = 0.996, *E* = 107.7%), and CDV (*Y* = −3.253*X + 39.974, *R*^2^ = 1.000, *E* = 103.0%), indicating good *R*^2^ and *E* for each virus in the multiplex real-time PCR.

### 3.3 Sensitivity of the multiplex real-time PCR assay

**Figure 3:**
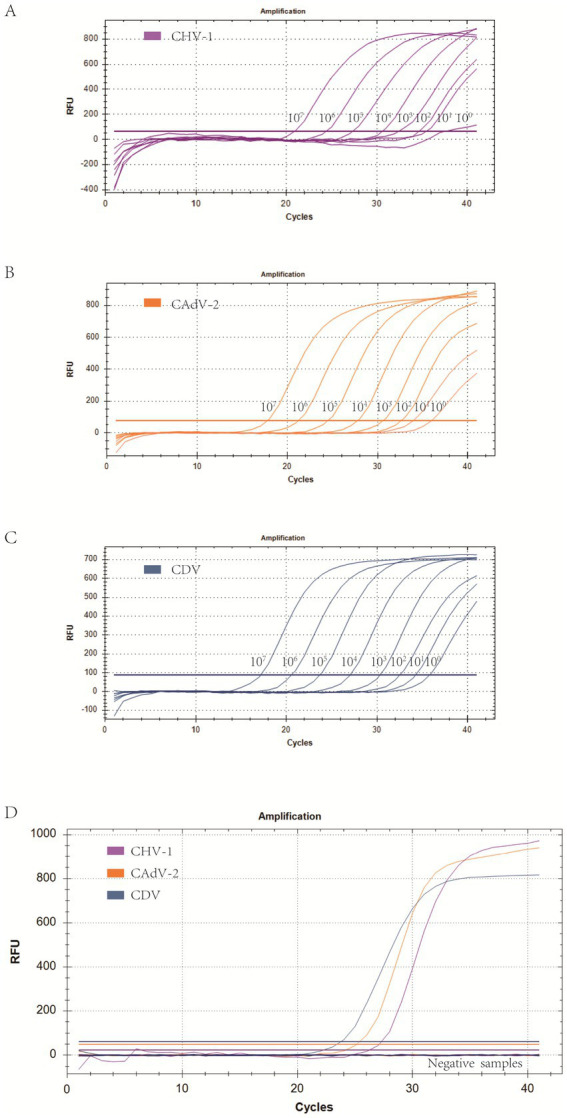
**(A–C)** Amplification curves (X-axis: Cycle, Y-axis: Fluorescence) of 10-fold serial dilutions (1 × 10^7^–1 × 10^0^ copies/μL) of plasmid standards for CHV-1, CAdV-2, and CDV detected by the multiplex real-time PCR assay. **(D)** Three amplification curves represent samples positive for CHV-1, CAdV-2, and CDV detected by our multiplex real-time PCR assay; negatives include CPV, CCoV, CPIV, CAstV, ChPV, and the negative control.

Using the optimized system and the plasmid standards of each pathogen with concentrations ranging from 1 × 10^7^ copies/μL to 1 × 10^0^ copies/μL, the results demonstrated that the method could identify CHV-1 positive samples with concentrations as low as 1 × 10^1^ copies/μL, while CAdV-2 and CDV positive samples could be detected with concentrations as low as 1 × 10^0^ copies/μL (Figures 3A–C). However, in follow-up experiments, the preliminary detection line was set at a Cq value = 35, and a Cq ≤ 35 was considered positive. The experimental results indicated that the detection rate of CHV-1 samples at 1 × 10^1^ copies/μL and CAdV-2, and CDV samples at 1 × 10^0^ copies/μL were less than 95% in replicates (Table 2). Thus, the reliable limit of detection for this method was 1 × 10^2^ copies/μL for CHV-1, 1 × 10^1^ copies/μL for CAdV-2, and 1 × 10^1^ copies/μL for CDV. The cutoff line for positivity was automatically determined by the instrument.

**Table 2:** Sensitivity results of multiplex real-time PCR.

Pathogens	Concentration (copies/uL)	Total	Positive (Cq ≤ 35)	Detection rate	Cq value range	95% detection rate
CHV-1	10^2^	25	25	100%	100%, <34.9	>95%
	10^1^	25	20	72%	72%, <35; 28%, <37.5	<95%
	10^0^	25	10	50%	50%, <35; 50%, <37.5	<95%
CAdV-2	10^1^	25	25	100%	100%, <34.8	>95%
	10^0^	25	20	84%	84%, <35; 16%, <35.6	<95%
	10^−1^	25	16	64%	64%, <35; 26%, <35.7	<95%
CDV	10^1^	25	25	100%	100%, <34.8	>95%
	10^0^	25	6	24%	24%, <35; 76%, <36.2	<95%

### 3.4 Specificity of the multiplex real-time PCR assay

To eliminate false positives caused by other viruses, CHV-1, CAdV-2, CDV, CPV, CCoV, CPIV, CAstV, and ChPV were all detected by the multiplex assay. The results showed that CHV-1, CAdV-2, and CDV, could be detected, while CPV, CCoV, CPIV, CAstV, and ChPV could not be successfully detected (Figure 3D), thus, indicating that the specificity of the method was excellent.

### 3.5 Repeatability of the multiplex real-time PCR assay

We chose different plasmid standards as the templates to test the C.V. of the method. The results showed that within different detection groups, C.V. values were below 2.26%, while those between different detection groups were below 1.33% (Table 3). Thus, those data indicated that the method has good stability.

**Table 3:** Validation of the detection repeatability of the developed multiplex real-time PCR method.

Pathogens	Concentration (copies/uL)	Intra-group test		Inter-group test	
		Cq ^a^ (Mean ±SD)	C.V. ^b^	Cq ^a^ (Mean ±SD)	C.V. ^b^
CHV-1	10^6^	24.83 ± 0.30	1.19%	24.55 ± 0.33	1.33%
	10^4^	29.86 ± 0.68	2.26%	29.83 ± 0.29	0.98%
	10^2^	34.51 ± 0.23	0.68%	34.31 ± 0.18	0.51%
CAdV-2	10^6^	21.60 ± 0.06	0.29%	21.46 ± 0.19	0.86%
	10^4^	28.00 ± 0.09	0.32%	28.02 ± 0.46	0.16%
	10^2^	32.68 ± 0.38	1.17%	32.75 ± 0.43	1.32%
CDV	10^6^	18.88 ± 0.18	0.98%	19.23 ± 0.13	0.66%
	10^4^	26.53 ± 0.07	0.25%	26.64 ± 0.26	0.96%
	10^2^	32.77 ± 0.18	0.56%	32.75 ± 0.23	0.69%

a Cycle quantification.

b Coefficient of variation.

### 3.6 Co-infection simulation experiment

**Figure 4:**
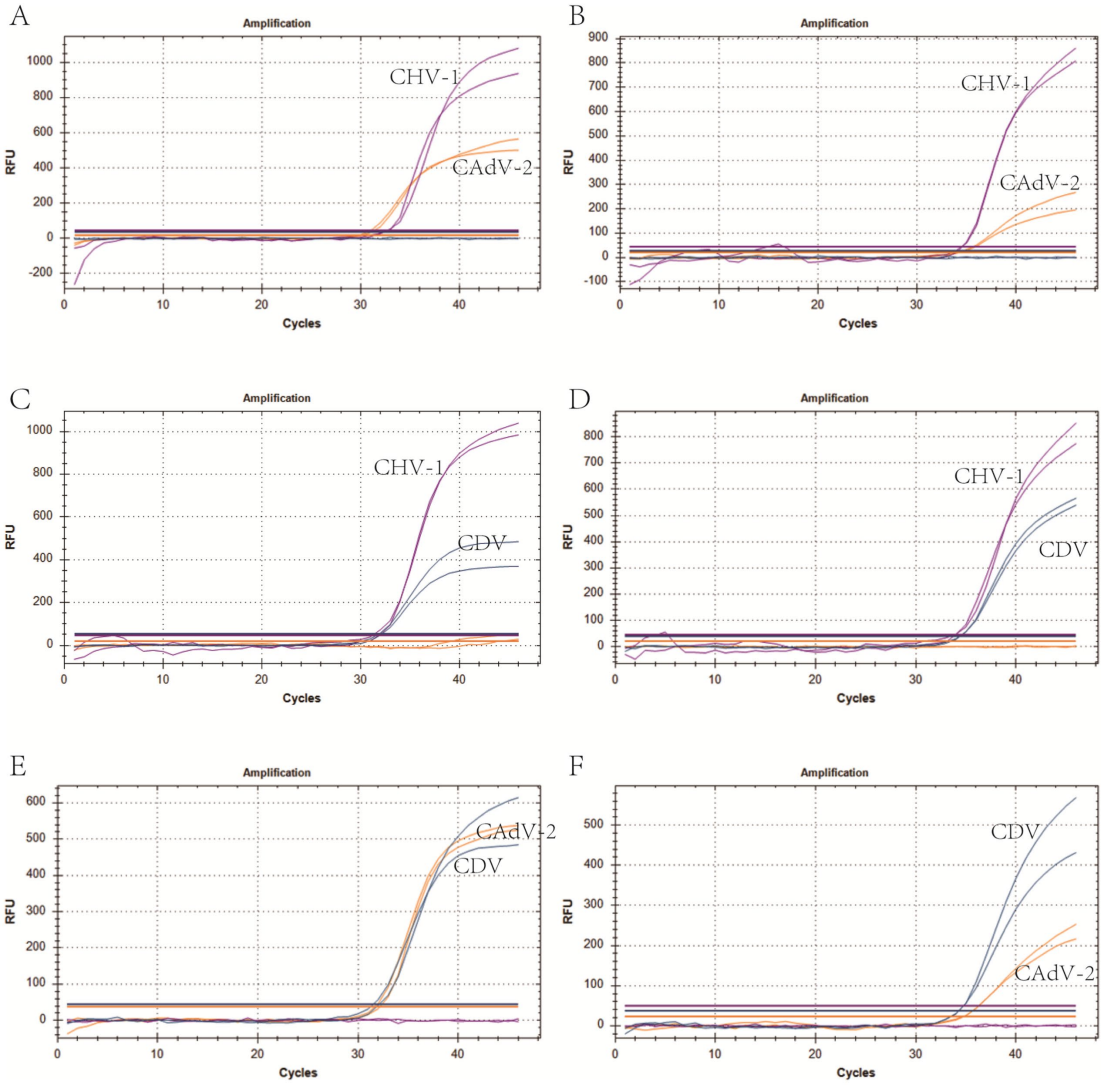
Co-infection simulation experiments with two pathogens. **(A,C,E)** Amplification curves (X-axis: Cycle, Y-axis: Fluorescence) of CHV-1 + CAdV-2, CHV-1 + CDV, CAdV-2 + CDV at concentrations of 10 times the limit of detection. **(B,D,F)** Amplification curves of CHV-1 + CAdV-2, CHV-1 + CDV, CAdV-2 + CDV at the limit of detection. Two replicates were used per reaction.

**Figure 5:**
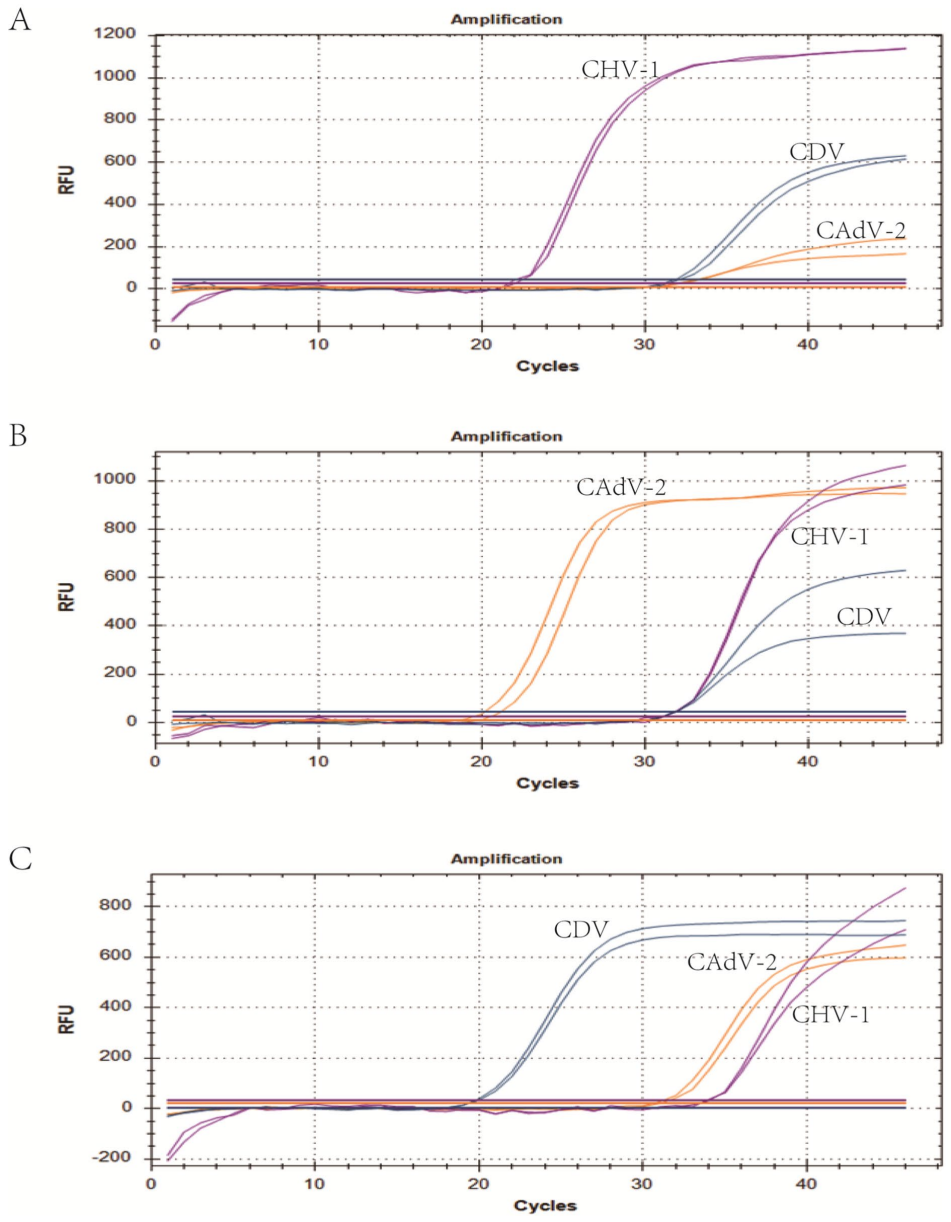
Co-infection of all three pathogens at different concentrations. **(A)** The concentration of plasmid standards for CHV-1 was 1 × 10^7^ copies/μL, while the others were 10 times the limit of detection. **(B)** The concentration of plasmid standards for CAdV-2 was 1 × 10^7^ copies/μL, while the others were 10 times the limit of detection. **(C)** The concentration of plasmid standards for CDV was 1 × 10^7^ copies/μL, while the others were 10 times the limit of detection. Two replicates were used per reaction. X-axis: Cycle; Y-axis: Fluorescence.

We selected plasmid standards with concentrations of 1 × 10^7^ copies/μL, the limit of detection, and 10 times the limit of detection of different pathogens as the templates on which to perform co-infection simulation experiments. The results showed that the multiplex method could detect simulated duplex co-infections with low concentrations (Figure 4), and simulated triple co-infections with different concentrations (Figure 5).

### 3.7 Clinical sample detection

To further validate the performance of the assay in clinical use and compare it to conventional PCR, 122 canine respiratory tract samples were collected and detected by conventional PCR and the method developed in this study. As shown in Table 4, for the three viruses, the conformance rates detected by the two methods were 97.54, 99.18 and 91.80%. The detection rates of CHV-1, CAdV-2, and CDV were 2.5, 6.3, and 13.9%, respectively. Additionally, the detection rate of CIRDC caused by the three viruses was 18% (22/122), while the co-infection rate was 22.7% (5/22). Five samples were found to contain two pathogens, including two co-infections with CHV-1 and CDV, and three co-infections with CAdV-2 and CDV. Those above results demonstrate that the developed assay had an excellent ability to detect co-infections.

**Table 4:** Clinical sample detection.

Pathogens	Pathogens		Conventional PCR		Conformance rate
			Positive	Negative	
Multiplex qPCR	CHV-1	Positive	0	3	97.54%
		Negative	0	119	
	CAdV-2	Positive	6	1	99.18%
		Negative	0	115	
	CDV	Positive	7	10	91.80%
		Negative	0	105	

## 4 Discussion

CIRDC is a globally prevalent infectious disease in canines. The pathogens involved in CIRDC include CAdV-2, CDV, and CHV-1, which have a high detection rate in dogs. Studies have shown that the symptoms of CAdV-2 and CDV are severe, while those of CHV-1 are more likely to show latent infection or aggravate respiratory symptoms in co-infections. Due to the transmission characteristics of the disease, it often occurs in large outbreaks, causing serious harm to related production and life (1, 7). Therefore, to more effectively control infections and mass outbreaks, reliable and practical early diagnosis has received increasing attention.

Currently, real-time PCR assays based on TaqMan probes are considered the most sensitive and specific detection methods (52). In this study, we developed a triple real-time PCR detection method based on TaqMan probes, which can simultaneously detect three viruses at different viral loads. The Cq values of each run represent the viral load, while different fluorescent markers distinguish the viruses.

The primers and probes are the key elements for developing novel triple real-time PCR detection methods, and their correct sequence ensures accurate target amplification and reliable fluorescence signal detection. All such factors are critical for successful detection. In this study, suitable primers and probes were designed and synthesized. The gB gene of CHV-1 is a common target in molecular diagnostic techniques and has been selected in a number of studies (31–33). In this study, primers and probes for CHV-1 were selected based on a prior report (49). The primers and probes for the detection of the CAdV-2 and CDV are specially designed and screened on target genes that are both conserved and specific. In terms of the selection of target genes, CAdV-2 selects the fiber gene sequence, which has high specificity and not only indicates CAV infection, but also distinguishes CAdV-2 from CAdV-1 using primers in the E3 region of the gene sequence (16, 53). CDV selects the N gene sequence. The H and F genes of CDV exhibit a high level of nucleotide variation, which allows the distinction of multiple genotypes or genetic clusters based on geographic patterns (24). Therefore, the selection of primers and probes was limited to conserved N-protein coding genes that exhibit low variability (25). After sequence selection, the conserved regions were identified by comparing the gene sequences of each virus. Primers and probes were designed based on the conserved regions, and their specificity was confirmed using NCBI BLAST. In addition, in multiplex real-time PCR assays, the concentration of primers and probes is used to determine the sensitivity and overall success of the assay by affecting the specificity of amplification and signal intensities. Therefore, meticulous optimization of primers and probe concentrations is imperative to achieve the optimal reaction efficiency. Ultimately, by considering the optimal reaction efficiency and their economic applicability, we optimized the parameters. The optimal volumes of primers for CHV-1, CAdV-2, and CDV were 0.8 μL (10 μM), 0.3 μL (10 μM), and 0.3 μL (10 μM), respectively. Similarly, the optimal volumes of probes were 0.8 μL (10 μM), 0.3 μL (10 μM), and 0.3 μL (10 μM), respectively.

The results of the plasmid standard curves demonstrated a high level of sensitivity for all primer-probe sets. As shown in Figure 2, the coefficient of determination (*R*^2^) exceeded 0.99 within the concentration range of 10^7^ to 10^2^ copies/μL, confirming the robust quantitative performance of the method. In sensitivity tests, the Cq limit was set to 35, and the lowest copy number that had at least a 95% probability of meeting the Cq limit in repeated experiments was considered the limit of detection. First, the maximum Cq values corresponding to a 100% positive detection rate for CHV-1, CAdV-2, and CDV were 34.9 (10^2^ copies/μL), 34.8 (10^1^ copies/μL), and 34.8 (10^1^ copies/μL), respectively. Second, the positive detection rate of CHV-1 of 10^1^ copies/μL was 72%, and the positive detection rates of CAdV-2 and CDV of 10^0^ copies/μL were 84 and 24%, respectively. For the smaller copy numbers, CHV-1 of 10^0^ copies/μL and CAdV-2 of 10^−1^ copies/μL, lower positive detection rates of 50 and 64% were observed. Therefore, the limit of detection of CHV-1, CAdV-2, and CDV were 10^2^ copies/μL, 10^1^ copies/μL, and 10^1^ copies/μL, respectively (Table 2). Compared to other detection methods for these viruses, the assay developed in this study is more sensitive to viral nucleic acids. In the singleplex real-time PCR detection method developed by Sui et al., the limit of detection of CDV was 22.5 copies/μL (54). Likewise, other conventional multiplex real-time PCR assays showed no noticeable advantage in terms of sensitivity (10). Hao et al. developed a multiplex PCR assay for canine respiratory viruses, in which the limit of detection was 1 × 10^4^ copies/μL (55).

The specificity of the triple real-time PCR assay was evaluated using other common pathogens that potentially infect canines. The results indicated that primers and probes in the assay were neither cross-reactive among the three viruses nor elicited nonspecific reactions with other common canine pathogens (Figure 3D). We used plasmids as the templates to test the C.V. values of the method. The results indicated that at high, medium, and low concentrations, the intra-group C.V. values were below 2.26%, while those between different detection groups were below 1.33%, indicating that the developed method possessed good stability (Table 3). The co-infection model gaged the detection efficiency of co-infections, confirming that the method could detect co-infections of two or three viruses simultaneously. Figure 4 shows that in a co-infection system with two viruses, the method obtained accurate and repeatable results, even when the concentration of the two viruses was close to the limit of detection (the concentration was tested at the limit of detection and 10 times the limit of detection). Figure 5 shows that when three viruses were in the co-infection model, the method exhibited effective and repeatable detection, even when the concentrations of several viruses in the sample were different (10^5^ copies/μL). The findings suggest that the triple real-time PCR method was accurate and applicable for analyzing clinical samples from canines affected by CIRDC.

We compared the detection results of the triple real-time PCR method developed in this study with conventional singleplex PCR methods (according to former reports or national standards) for clinical sample detection. These findings indicate that the two methods have a relatively high conformance rate for the detection of the three viruses (97.54% for CHV-1, 99.18% for CAdV-2, and 91.81% for CDV), and only a few positive samples with relatively high Cq values were detected as negative using the conventional PCR method (Table 4). Therefore, compared to conventional PCR, the method developed in this study is suitable for the clinical detection of CIRDC. Previous studies have reported that among dogs, especially those with respiratory symptoms, the detection rates of CAdV-2 and CDV viruses were relatively high, reaching 21 and 14%, respectively (4, 56). Conversely, the positivity rate for CHV-1 was relatively low, including 4% in one report, which was lower than the 9% positivity rate for CAdV-2 in this report (57). Those findings are consistent with our clinical test results: the positive detection rate of CHV-1 (2.5%) was slightly lower than the positive detection rates of CAdV-2 (6.3%) and CDV (13.9%). The positivity rate in our study was lower than that of other studies, which may be due to the differences in sample collection area and the collection season. In addition, co-infections were also common in previous studies, including co-infections of CAdV-2 and CDV (9, 58, 59), and CHV-1 and CDV (10). Co-infections of CAdV-2 and CDV are more common than co-infections of CHV-1 and CDV (10), which is consistent with our results. The frequent occurrence of co-infection also emphasizes the importance of using multiple methods to monitor CIRDC pathogens. The method established in this study is applicable for the detection CIRDC pathogens in canine populations. This is not only because the detection of the three main CIRDC pathogens was sensitive and reliable, but also because the method is simple to perform and economic, making it suitable for large-scale detection. Moreover, the samples detected using this method included blood, feces, and nasopharyngeal swabs, which is convenient in clinical sampling.

Multiplex real-time PCR has better and faster detectability with lower labor costs compared to conventional singleplex PCR, but its development is challenging due to technical difficulties at the development stage. In this study, the sensitivity of CHV-1 in the triple detection method was lower than that of the other two viruses, which may be due to the mutual interference among primers and probes. In addition, there are some limitations to this study. The insufficient number of clinical samples tested in this study affects its further application in large-scale studies. At the same time, the study only compared the new assays to conventional PCR and lacked comparisons to antigen detection methods. In future studies, we will collect additional clinical samples and compare them using multiple detection methods to provide more powerful evidence for the application of this method in large-scale clinical detection.

In short, an efficient detection method will be beneficial for the monitoring and prevention of CIRDC. To the best of our knowledge, this is the first multiplex real-time PCR detection method for CHV-1, CAdV-2, and CDV, which often present simultaneously in CIRDC. The multiplex detection method can detect multiple pathogens in a single reaction, making the detection of co-infections more convenient than is currently possible. This method also exhibits good specificity and sensitivity, which reduces labor and material costs.

## 5 Conclusion

This study successfully established a multiplex real-time PCR method based on TaqMan probes for the simultaneous detection of three viruses associated with CIRDC in dogs. The method exhibited efficient detection capabilities, and excellent specificity and repeatability. The method is suitable for clinical differential diagnosis of co-infections and facilitates the early diagnosis and treatment of clinical cases. Moreover, if the method can be used for large-scale surveillance, it will also be of great significance for epidemiological research on CIRDC.

## Data Availability

The original contributions presented in the study are included in the article/supplementary material, further inquiries can be directed to the corresponding author.

## Footnotes

^1^https://blast.ncbi.nlm.nih.gov/Blast.cgi?PROGRAM=blastn&PAGE_TYPE=BlastSearch&LINK_LOC=blasthome, accessed on 1 September 2023.
